# Clinical Rehabilitation Effect of Postoperative Lower-Limb Training on the Patients Undergoing OLIF Surgery: A Retrospective Study

**DOI:** 10.1155/2020/1065202

**Published:** 2020-01-16

**Authors:** Haoming Wang, Yachong Huo, Yachao Zhao, Botong Zhang, Dalong Yang, Sidong Yang, Wenyuan Ding

**Affiliations:** ^1^Department of Spine Surgery, The Third Hospital of Hebei Medical University, 139 Ziqiang Road, Shijiazhuang 050051, Hebei Province, China; ^2^Hebei Provincial Key Laboratory of Orthopaedic Biomechanics, 139 Ziqiang Road, Shijiazhuang 050051, Hebei Province, China

## Abstract

**Background:**

In this study, it was aimed to investigate the clinical rehabilitation effect of lower-limb training on the patients that undergo oblique lumbar interbody fusion (OLIF) procedures.

**Methods:**

The eligible participants undergoing OLIF procedures between 01/2017 and 07/2019 were identified. All the patients underwent one-segment fusion operation (L3-4 or L4-5). Based on whether the participants received postoperative rehabilitation training, they were divided into two groups: intervention group and control group. Postoperatively, the participants in the intervention group were trained with lower-extremity rehabilitation exercise and maintained for three months. All participants got reexamined at the first postoperative week, the second postoperative week, the first postoperative month, and the third postoperative month (last follow-up). Comparisons were made in terms of the lower-extremity muscle force, visual analogue scale (VAS) score, lumbar JOA score, Oswestry disability index (ODI), the incidence of deep venous thrombosis (DVT), and patient satisfaction.

**Results:**

Seventy-seven participants in the intervention group (32 males and 45 females) and 82 in the control group (39 males and 43 females) were incorporated in this study. The median age of the participants was 57 years (39∼73) in the intervention group and 54 years (35∼71) in the control group. No statistical significance between the two groups was found (*P* > 0.05). ODI score was less in the intervention group as compared to the control group in the first week after surgery (*P*=0.029). VAS and JOA scores were better in the intervention group in the first two weeks after surgery (*P* < 0.05). DVT incidence in the intervention group was lower than the control group at final follow-up (*P*=0.037). Both group participants have achieved good grading in muscle force rehabilitation but no significant differences between the two groups. Additionally, satisfaction was higher in the intervention group than the control group.

**Conclusions:**

In summary, postoperative lower-extremity rehabilitation exercise can effectively accelerate patients' health recovery from the OLIF surgery and increase their satisfaction.

## 1. Introduction

Clinically, intervertebral disc degeneration (IVDD) is not uncommon, and its derived diseases, such as lumbar disc herniation, are prevalent in spine departments; interbody fusion surgeries are widely accepted and used in treating IVDD-associated diseases, particularly for lumbar and cervical spinal diseases [1–3]. Although the surgical techniques have much advanced, the patients would have worse health outcomes (physically and mentally) after undergoing lumbar interbody fusion (LIF) surgeries, as compared with the general population [4–6]. Furthermore, many patients (even up to 40%) after fusion surgeries reported that they persistently suffered from lower back pain (LBP), functional disability, and poor life quality; around 20% of the patients would return to reoperations [7–10]. Thus, the patients were often required by the surgeons to perform physical therapy treatment to accelerate the rehabilitation process after spinal operations. Noticeably, the findings from previous studies add to the growing body of evidence that postoperative rehabilitation training has improved health outcome after spinal disc surgeries, and a high-intensity rehabilitation training program is more likely to relieve the pain and decrease the disability events than a low-intensity rehabilitation training program [11–13].

As we know, oblique lumbar interbody fusion (OLIF), different from the anterior lumbar interbody fusion (ALIF), is an extraperitoneal approach that has a lower incidence of vascular injury and abdominal complications, as well as the reverse ejaculation [14, 15]. However, some other complications, for instance, the deep venous thrombosis (DVT) and atrophy of the lower-extremity muscle possibly occur and exist after OLIF surgeries. In our spine departments, as a prophylaxis procedure of the complications, the patients were routinely asked to practise systematic lower-limb rehabilitation procedures postoperatively, as we reported previously [16, 17], and maintained the same intensity rehabilitation training programs for 3 months. However, not all the patients followed our clinical guidance, and they might have undergone a different recovery process from others.

Therefore, in this retrospective study, we aimed to investigate the postoperative clinical rehabilitation effect of the systematic lower-limb exercise on the patients that underwent OLIF procedures, for the purpose to better understand the postoperative rehabilitation procedures.

## 2. Methods

### 2.1. Statement of Ethical Approval

Our current study was conducted with an ethical approval from the local medical ethics council affiliated to our hospital, and the informed consent forms have been reviewed and signed by the patients or their lawful guardians.

### 2.2. Participants

**Figure 1:**
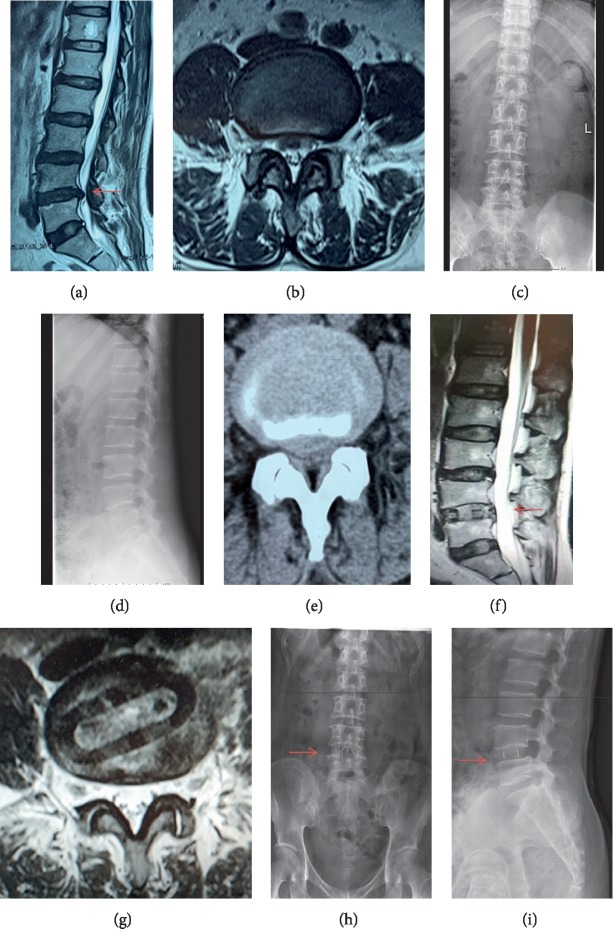
A representative case of OLIF surgery with radiographs and MRI scan: (a, b) preoperative MRI scan, (c, d) preoperative X-ray radiographs, (e) preoperative axial CT scan, (f, g) postoperative MRI scan, and (h, i) postoperative X-ray radiographs.

The eligible participants undergoing OLIF procedures between 01/2017 and 07/2019 were identified, and their medical records were retrospectively collected. Figure 1 shows a case as a representative of OLIF surgery. Based on whether the participants received postoperative rehabilitation training, they were divided into two groups: intervention group and control group. During the period of perioperation, all the participants in both groups had undergone the same routine medical care, as well as chemical prophylaxis with low-molecular-weight heparin after surgery. Postoperatively, the participants in the intervention group were trained with systematic lower-limb rehabilitation procedures as we reported previously [16, 17] and maintained for 3 months. All participants got reexamined at the first postoperative week, the second postoperative week, the first postoperative month, and the third postoperative month (last follow-up).

### 2.3. OLIF Procedures

Regarding the surgical procedures, the patients in the left lateral position underwent general anesthesia. The OLIF procedures were carried out subsequently as follows: marking incision, blunt dissection of muscle, application of retractor, and then discectomy; a stand-alone cage of suitable size was inserted into the intervertebral disc space, as shown in Figures 1(h) and 1(i). The surgical segments were L3-4 or L4-5. All the patients underwent one-segment fusion operation, and no posterior internal fixation was performed in the OLIF surgery.

### 2.4. Clinical Assessment

Clinical comparisons were made in terms of the muscle force of lower limbs, visual analogue scale (VAS) score, lumbar Japanese Orthopaedic Association score (JOA score, 29 points), Oswestry disability index (ODI), lower-extremity deep venous thrombosis (DVT), and patient satisfaction. The muscle force grading of lower limbs was performed with reference to the classification criteria that the British medical research council applies. Additionally, the satisfaction rate was calculated with three levels: dissatisfied, satisfied, and very satisfied.

### 2.5. Statistical Analyses

In this study, the software SPSS for Windows 21.0 (SPSS Inc., USA) was utilized for statistical analysis. The data of VAS score, JOA score, and ODI are shown with mean ± standard deviation (SD). The data of age are shown with median. Multiple comparisons were conducted using analysis of variance after the parameters were verified to accord with the homogeneity and normality of variance, and subsequently Student's *t*-test was carried out with Bonferroni correction. The chi-square tests were performed to statistically analyze the count data, including baseline data, muscle strength, DVT incidence, and the patient satisfaction. *P* value standing for significance was set as 0.05.

## 3. Results

### 3.1. Baseline Demographical Data

**Figure 2:**
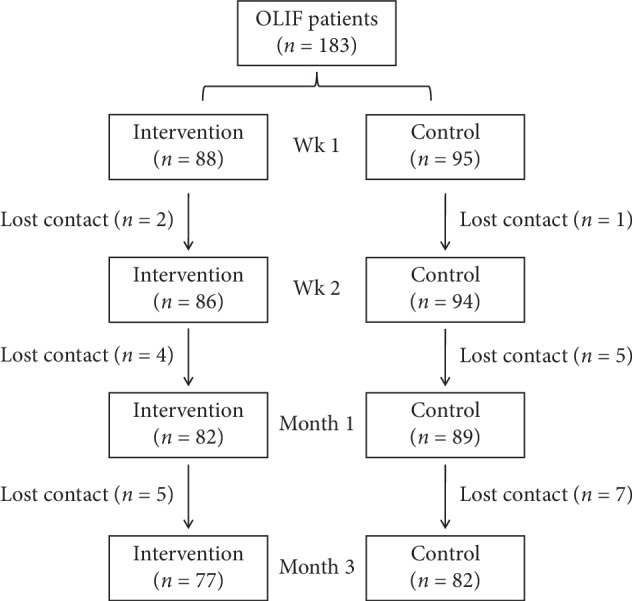
Schematic diagram of this study.

As shown in Figure 2, after the identification of participant eligibility, 159 participants were finally incorporated in this study, including 77 cases in the intervention group and 82 cases in the control group. The intervention group consists of 32 males and 45 females, and the control group consists of 39 males and 43 females. The median age of the participants is 57 years (39∼73) in the intervention group and 54 years (35∼71) in the control group. Statistical analysis of the baseline data showed no significance between the two groups mentioned above (*P* > 0.05).

### 3.2. Muscle Strength Grading of the Lower Limbs

Compared with the preoperative grading, the participants in both groups have achieved better grades in terms of muscle strength rehabilitation, but no significant differences can be detectable between the two groups regardless of the time points (Table 1).

### 3.3. VAS Score

As shown in Table 2, both groups have obtained better scores as compared with the preoperation. In addition, less VAS scores have been achieved in the intervention group than the control group at the time points of the first and second week after surgery (*P*=0.042 and *P*=0.001, respectively). However, no detectable significances exist at the first postoperative month or the third postoperative month (*P* > 0.05).

### 3.4. JOA Score

As shown in Table 3, both groups have obtained better JOA scores as compared with the preoperation. Notably, more JOA scores have been achieved in the intervention group than in the control group at the time points of the first two weeks after surgery (*P*=0.004 and *P* < 0.001, respectively). However, no detectable significances exist at the first postoperative month or the third postoperative month (*P* > 0.05).

### 3.5. ODI Score

As we can see in Table 4, both groups have obtained significant improvement of the ODI score as compared with the preoperation. Clearly, ODI scores are less in the intervention group than in the control group in the first week after surgery (*P*=0.029). However, no detectable significances exist at the second postoperative week, the first postoperative month, or the third postoperative month (all *P* > 0.05).

### 3.6. DVT Incidence

Table 5 shows that lower-limb DVT events have been examined at the first postsurgery week and the third postsurgery month (last follow-up). As a result, DVT incidence in the intervention group is lower than in the control group at final follow-up (*χ*^2^=4.354, *P*=0.037).

### 3.7. Patient Satisfaction

Table 6 is a summary of patient satisfaction. As we can see, the differences of patient satisfaction are insignificant between the intervention group and the control group in the first postsurgery week (*P* > 0.05). However, the patients in the intervention group are more satisfied about the treatment than those in the control group when they are compared at the second postoperative week, the first postoperative month, and the third postoperative month (*χ*^2^=14.15, *P*=0.001; *χ*^2^=12.64, *P*=0.002; and *χ*^2^=8.025, *P*=0.018, respectively).

## 4. Discussion

It is reported that OLIF surgery has a lower incidence of vascular injury and abdominal complications, as well as the reverse ejaculation [14, 15]. However, some other complications, for instance, the deep venous thrombosis (DVT) and atrophy of the lower-extremity muscle, might occur and exist after OLIF surgeries. In the spine department of our hospital, the patients are routinely asked to learn how to practise systematic lower-limb rehabilitation procedures postoperatively and should maintain the same intensity rehabilitation training programs for 3 months. Surely, that is regarded as a prophylaxis procedure of the complications and has been verified effective in our previous studies [16, 17]. However, not all the patients are willing to follow our clinical guidance, and the patients that have not followed the guidance might have undergone a different recovery process from others. In this study, we therefore investigated the possible clinical rehabilitation effect of systematic lower-limb exercise on the patients that had undergone OLIF procedures, with the purpose of better understanding the postoperative rehabilitation management.

Finally, it comes to our attention that the participants in both groups have achieved better grades in terms of muscle strength rehabilitation, as compared with the preoperative grading, but no significant differences can be detectable between the two groups regardless of the time points. Clearly, our postoperative rehabilitation training has not made a change to the muscle strength as theoretically expected. It is speculated that it might be due to the small sample in this study. Yet, less VAS scores and more JOA scores have been achieved with the postoperative rehabilitation training at the time points of the first and second week after surgery although no detectable significances exist at the first postoperative month or the third postoperative month. Furthermore, better ODI scores are presented after postoperative rehabilitation training in the first postsurgery week.

Additionally, DVT events of the lower extremities have been examined at the first postsurgery week and the third postsurgery month (last follow-up). As a result, DVT incidence with postoperative rehabilitation training is significantly lower than the control group at final follow-up. As we can see from the patient satisfaction survey, the postoperative rehabilitation training did not make any differences regarding the patient satisfaction in the first postsurgery week, but the patients with postoperative rehabilitation training are more satisfied about the surgical treatment outcomes than those without postoperative rehabilitation training when compared at the second postoperative week, the first postoperative month, and the third postoperative month (the last follow-up). All the results we have obtained in this study suggest that postoperative rehabilitation training can improve the functional outcomes (better ODI and JOA score), relieve the pain (decreased VAS score), reduce the postoperative complications (decreased DVT incidence), and increase the patient satisfaction. Therefore, the findings in this study have added to the body of evidence that the systematic lower-limb rehabilitation training is positively effective to accelerate the postoperative health recovery after spinal OLIF operations.

To date, it has been controversial for a long time on whether the postoperative rehabilitation is effective to promote health recovery and decrease the complications after spinal surgeries. Some scientists [12, 18, 19] stand for it that postoperative rehabilitation would be positive on the health recovery after the patients undergo spinal operations, in terms of pain relief, functional improvement, shortening the length of hospital stay, and high patient satisfaction. However, some other investigators may have a different viewpoint, even contrary to those positive ones. They reported that postoperative rehabilitation management did not work as it should be, showing that the functional outcome, the disability, and patient life quality were not improved, even in a long-term observation [1, 20, 21]. Although the previous studies have drawn contradictory conclusions, our current study results are supporting the positive point of view that the postoperative rehabilitation is effective to promote health recovery and decrease the complications after spinal surgeries.

There are some study limitations in our current work. First of all, this is a single-center, retrospective, comparative study; thus, it is lacking extensive representativeness. Second, the blind method has not been employed throughout the whole study. In addition, it would be much better if the patient sample size is larger. As such, a preferred study in future should guarantee that all the deficiencies and flaws mentioned above are overcome, probably a prospective, multicenter, randomly controlled study, with a large sample size and blind methods utilized.

## 5. Conclusions

In summary, the lower-extremity rehabilitation exercise can effectively promote patient health recovery from the OLIF surgeries, characteristic of accelerated pain relief and improved functional outcomes, and also can be helpful to reducing DVT events of the lower limbs and achieve a higher patient satisfaction rate. Hence, the patients should be encouraged to perform and maintain the systematic lower-limb rehabilitation training after they undergo spinal OLIF surgeries.

## Figures and Tables

**Table 1:** Assessment and comparisons regarding muscle force of the lower extremities.

Group	Preoperation		Po-1^st^ wk			Po-2^nd^ wk			Po-1^st^ month			Po-3^rd^ month		
Grade	III	IV	III	IV	V	III	IV	V	III	IV	V	III	IV	V
Control (*n* = 82)	35	47	18	52	12	9	45	28	2	10	70	0	2	80
Intervention (*n* = 77)	29	48	13	55	9	6	41	30	1	8	68	0	2	75

*P* < 0.001, in terms of muscle force comparison between preoperation and postoperation. Po-, postoperation.

**Table 2:** VAS score assessment and comparisons.

Group	Preoperative	1^st^ wk	2^nd^ wk	1^st^ month	3^rd^ month
Control (*n* = 82)	6.5 ± 2.0	4.0 ± 2.2	2.8 ± 1.2	1.7 ± 1.3	1.0 ± 0.4
Intervention (*n* = 77)	6.2 ± 2.2	3.3 ± 2.1	2.2 ± 1.1	1.5 ± 1.3	1.0 ± 0.3
*P* value	0.369	*0.042*	*0.001*	0.334	>0.99

VAS score: visual analogue scale score.

**Table 3:** JOA score assessment and comparisons.

Groups	Preoperative	1^st^ wk	2^nd^ wk	1^st^ month	3^rd^ month
Control (*n* = 82)	7.8 ± 1.2	12.5 ± 2.1	15.6 ± 2.7	19.5 ± 6.0	23.0 ± 6.5
Intervention (*n* = 77)	8.0 ± 1.4	13.4 ± 1.8	17.8 ± 2.6	20.0 ± 7.0	23.5 ± 6.5
*P* value	0.334	*0.004*	*<0.001*	0.629	0.628

JOA: Japanese Orthopaedic Association.

**Table 4:** Comparisons in terms of ODI.

Groups	Preoperative	1^st^ wk	2^nd^ wk	1^st^ month	3^rd^ month
Control (*n* = 82)	45 ± 21	34 ± 21	29 ± 14	17 ± 12	10 ± 5
Intervention (*n* = 77)	44 ± 23	27 ± 19	27 ± 15	15 ± 13	10 ± 6
*P* value	0.775	*0.029*	0.386	0.315	>0.99

ODI: Oswestry disability index.

**Table 5:** The assessment and comparisons in terms of DVT events.

Group	Po-1^st^ wk		Po-3^rd^ month	
	DVT	Non-DVT	DVT	Non-DVT
Control (*n* = 82)	13	69	7	75
Intervention (*n* = 77)^*∗*#^	7	70	1	76

^*∗*^ *χ* ^2^=1.652, *P*=0.199; ^#^*χ*^2^=4.354, *P*=0.037, in comparisons at the first postsurgery week and the third postsurgery month (last follow-up), respectively. DVT, deep venous thrombosis; Po-, postoperation.

**Table 6:** Comparisons in terms of patient satisfaction rate.

After surgery	Intervention group (*n* = 77)	Control group (*n* = 82)	Chi-square tests	
	Very satisfied/satisfied/dissatisfied	Very satisfied/satisfied/dissatisfied	*χ* ^2^	*P* value
Wk 1	17 cases/56 cases/4 cases	9 cases/65 cases/8 cases	4.311	0.116
Wk 2	45 cases/30 cases/2 cases	24 cases/52 cases/6 cases	*14.15*	*0.001*
Month 1	58 cases/19 cases/0 cases	40 cases/40 cases/2 cases	*12.64*	*0.002*
Month 3	65 cases/12 cases/0 cases	54 cases/26 cases/2 cases	*8.025*	*0.018*

## Data Availability

Data are available regarding this study.
